# Bacteriophages targeting *Enterococcus faecalis* strains - a potential new root canal therapy

**DOI:** 10.1080/20002297.2017.1325237

**Published:** 2017-07-10

**Authors:** Mohammed Al Zubidi

**Affiliations:** ^a^ School of Clinical Dentistry, University of Sheffield, Sheffield, UK

## Abstract

The most common isolated species from recalcitrant endodontic infections is *Enterococcus faecalis*, a gram positive multiply drug-resistant and environmentally stable bacterium. As a potential route to a biological solution to *E. faecalis* root-canal infection we aimed to isolate, characterise and test *in vitro* a range of lytic bacteriophages targeted against *E. faecalis* isolates from oral endodontic infections obtained from labs across Europe.

Five bacteriophages were isolated from concentrated waste water named (SHEF2,4,5,6,7) that belong to the Siphoviridae family. They are specific to *E. faecalis* with genome sizes ranging from 39-43kb. Full chromosome sequences of SHEF2,4 and 5 alongside biological evidence revealed that they are lytic bacteriophages which place them as suitable candidate for therapy. We tested the ability of these phages to eradicate biofilm from abiotic surfaces and a novel cross-sectional tooth model while also showing that they are able to rescue Zebrafish embryos from *E. faecalis* systemic clinical strain infection. Finally, we established that the extracellular exopolysaccharide of *E. faecalis* was the bacterial docking target of these phages during the initial stages of phage infection. We suggest that these or other bacteriophages might be novel adjuncts to current endodontic therapy to eradicate recalcitrant biofilm and antibiotic resistant *E. faecalis.*

